# Moderate Sun Exposure Is the Complementor in Insufficient Vitamin D Consumers

**DOI:** 10.3389/fnut.2022.832659

**Published:** 2022-03-08

**Authors:** Shou-En Wu, Wei-Liang Chen

**Affiliations:** ^1^Department of Dermatology, Tri-Service General Hospital, School of Medicine, National Defense Medical Center, Taipei City, Taiwan; ^2^Division of Family Medicine, Department of Family and Community Medicine, Tri-Service General Hospital, School of Medicine, National Defense Medical Center, Taipei City, Taiwan; ^3^Division of Geriatric Medicine, Department of Family and Community Medicine, Tri-Service General Hospital, School of Medicine, National Defense Medical Center, Taipei City, Taiwan; ^4^Department of Biochemistry, National Defense Medical Center, Taipei City, Taiwan

**Keywords:** sun exposure, vitamin D, dietary intake, dietary supplements, vitamin D deficiency

## Abstract

**Background and Aims:**

Vitamin (Vit) D plays a vital role in human health, and the prevalence of Vit D deficiency worldwide has been a rising concern. This study investigates the serum 25-hydroxy-Vit D [25(OH)D] status in healthy US civilians and identifies how the two main sources, sun exposure and dietary Vit D intake, determine the final 25(OH)D levels in individuals.

**Methods:**

A total of 2,360 of participants from The National Health and Nutrition Examination Survey (NHANES) 2009–2014 were analyzed. We divided the levels of sun exposure and dietary Vit D intake of all subjects into 10 strata and gave a score ranging from 1 to 10 points, respectively. Scores 1–5 in sun exposure and dietary intake were considered as relatively low exposure groups, whereas scores 6–10 were considered as relatively high exposure groups. Serum Vit D inadequacy was defined as <50 nmol/L. Linear and logistic regression analyses were used to examine the associations between sources of Vit D and serum 25(OH)D levels.

**Results:**

In relatively low Vit D intake groups (Vit D intake score 1–5), relatively high sun exposure (sun exposure score >5) resulted in higher serum 25(OH)D levels (average 57 nmol/L) compared with relatively low sun exposure (sun exposure score <5) (average 50 nmol/L), whereas this difference became negligible in high intake groups (Vit D intake score 6–10). Moreover, the greatest slope occurred in the low Vit D intake curve (decile 3 of Vit D intake), which shows increased sun exposure time causing the greatest degree of change in serum 25(OH)D level in this group.

**Conclusion:**

Sun exposure can significantly make up for deficiencies in subjects who consume insufficient dietary Vit D. Compared with the extra cost and time for building habits of Vit D supplementation, moderate sun exposure appears to be a simple and costless means for the public to start in daily practice.

## Introduction

Growing evidence on health effects brought by Vitamin (Vit) D in human has expanded from well-established benefits such as bone growth and calcium homeostasis (1) to protective roles in cardiovascular diseases (2), metabolic syndrome (3), autoimmune disorders (4), and malignancies (5). It reiterates the importance of the two main sources of Vit D, cutaneous photosynthesis, and diet, but also brings the long-lasting “Vit D controversy” to the fore (6). Scientists have argued about the risk and benefit in both parties. Abundant studies have illustrated the intake of Vit D gaining the upper hand, in the consideration of the carcinogenic property of excessive UV radiation, uncertain individual responsiveness, and the inaccessibility for countries at high latitudes (7, 8). Nevertheless, emerging studies are discussing the incomparable advantages of obtaining Vit D from sun exposure, which include the reduction in all-cause mortality (9, 10), cardiovascular benefits (11), and potential lower COVID-19-specific mortality (12). The call for transforming the concept of absolute sun avoidance to acceptance of non-burning sun exposure is grasping attention (13).

Recommendations for the two main sources of Vit D are not alike. Whereas diets are highly promoted with few restrictions for upper limits (14, 15), sun exposure is cautiously suggested with maximum dose of exposure (16, 17). Sufficient dietary Vit D mostly rely on fortified foods or dietary supplements due to the scarcity of Vit D in natural foods (18). On the contrary, UVB radiation required for sufficient Vit D synthesis can be easily achieved by approximately 5–30 min of sun exposure to arms and legs, particularly around midday (between 10 am and 4 pm), at least two times a week (18–20). The risk of skin malignancy rather imposes threat which prompts experts to advise photoprotective measures. In view of this, dietary source seems to attract more attention than sunlight in the recommendations for sufficient Vit D synthesis, but is this true and feasible in daily practice?

This study utilized representative data of US civilian, the National Health and Nutrition Examination Survey (NHANES) (21), to compare the impacts of sun exposure and dietary Vit D intake on serum total 25-hydroxy-Vit D [25(OH)D] level. The aim of our study is to present the realistic Vit D status in general US population and discuss the efficacies of the two resources to make recommendations that is feasible for adequate Vit D gain in daily practice.

## Materials and Methods

### Study Design and Participants

The National Health and Nutrition Examination Survey is a large and nationally representative survey which collects health and nutrition data of non-institutionalized civilian resident population of the US (21). NHANES consists of two parts which are household interviews followed by a standardized physical examination carried out in mobile examination centers (MECs). Detailed information of questionnaires, procedure manuals, and method descriptions is available on the official website.^1^

From the total participants of 11,842 in NHANES 2009–2014, we excluded those with missing information of sun exposure time, Vit D intake, and serum 25(OH)D level, leaving 2,360 eligible for further analysis. We compared the relationship between serum 25(OH)D level and two major sources of synthesis: sun exposure and dietary intake. To clarify the strengths and weaknesses of both, we present the sun exposure or dietary intake either in a continuous manner or divided into decile ranking. Ultimately, we added up the two variables to provide an integrated view of both resources and make feasible suggestions for daily practice.

### Measurement of Sun Exposure Time

Since NHANES 2009–2010 (21), participants were asked about the time [minutes(min)] they spent outdoors during the past 30 days between 9 am and 5 pm on work and non-workdays. We took the average value of the answers on work and non-workdays of each individual to present daily sun exposure time. Nonetheless, the same sun exposure time in different geographical locations and different seasons may result in widely differing doses of ultraviolet B (UVB). This is one of the limitations of NHANES study since we could only obtain sun exposure time instead of the accurate UVB exposure dosage.

### Measurement of Dietary Vitamin D Intake

The NHANES dietary interview estimates the energy, nutrients, and other food components from foods and beverages (21). USDA’s Food and Nutrient Database for Dietary Studies (FNDDS) was used for processing the intake data by coding each food type and portion and converting them into computable nutrient values^2^ (22). The NHANES data provided have been processed and released in collaboration with the corresponding FNDDS version (23). Supplement usage was acquired from a specific 24-h dietary supplement interview. We combined both dietary and supplementation Vit D data to calculate the total dietary Vit D intake data in this study.

### Calculation of Sun Exposure Time Score and Vitamin D Intake Score

To evaluate and compare the impact of sun exposure time and Vit D intake on serum 25(OH)D level, we divided the participants into 10 strata each of sun exposure time and Vit D intake. Participants having the lowest decile of sun exposure time and Vit D intake received 1 point, and the highest decile received 10 points. The corresponding values for the sun exposure and Vit D intake score are provided in Table 1. In the following analyses, scores 1–5 in both sun exposure and Vit D intake were considered as relatively low groups, whereas scores 6–10 were considered as relatively high groups.

**TABLE 1:** The corresponding values for the sun exposure time and vitamin D intake score.

Points	Daily sun exposure time			Daily vitamin D intake		
	N	Time(min)	Serum 25(OH)D level(nmol/L)	N	Amount(mcg)	Serum 25(OH)D level(nmol/L)
1	296	0	58	267	0	53
2	128	14	60	221	1	52
3	284	35	63	222	2	54
4	278	60	63	243	3	60
5	273	85	64	233	5	59
6	238	117	65	199	7	61
7	156	147	64	237	10	65
8	236	191	68	232	14	70
9	188	250	66	253	22	72
10	283	363	64	253	52	88

### Measurement of Serum 25(OH)D Level

Quantitative detection of serum Vit D level within the NHANES study has used the ultrahigh-performance liquid chromatography–tandem mass spectrometry method (Thermo Scientific, Carlsbad, CA, United States) starting from NHANES 2007–2008 (24). According to the manual of Centers for Disease Control and Prevention (CDC) laboratory, the total 25(OH)D concentration (nmol/L) was defined as the sum of 25-hydroxyVit D3 (25OHD3) and 25-hydroxyVit D2 (25OHD2) (25). This method improves analytical specificity and sensitivity comparing to the past and fixed analytical goals for imprecision (≤10%) and bias (≤5%).

### Definition of Vitamin D Inadequacy

Given that the study population of this study is US civilian, we adopted the definition suggested by expert committees of the Food and Nutrition Board (FNB). Vit D inadequacy was defined as serum 25(OH)D concentrations <50 nmol/L (26).

### Covariates

The formula of body mass index (BMI) was kg/m^2^ where kg is weight in kilograms and m^2^ is height in meters squared. Smoking history was considered positive by answering yes to the question “Have you smoked at least 100 cigarettes in your entire life?” Serum alanine aminotransferase and creatinine levels were determined using DcX800 kinetic rate method and the Jaffe rate method, respectively. These variables are included in adjusted models based on their correlations with Vit D status proposed in the previous literature. There are age, gender, and race differences with respect to Vit D status (27, 28). An inverse association is observed between Vit D status and obesity (29). Commonly suggested mechanisms included the decrease of sun exposure due to limited motility in obese individuals and sequestration of Vit D within the adipose tissue (30). Aside from BMI which represents total obesity, parameters of central obesity such as waist circumference and waist–hip ratio have also been demonstrated to be negatively related to Vit D level especially in women (31). These evidences suggest the importance of taking obesity into account in the evaluation of Vit D status. The metabolism pathway of activated Vit D involves liver and kidney (32), and patients with abnormal functions of both organs are at risk of Vit D deficiency (33, 34). Smoking is associated with lower level of circulating 25(OH)D (35).

### Statistical Analysis

Statistical analyses were performed using SPSS Complex Sample software (IBM Corp., Released 2013. IBM SPSS Statistics for Windows, version 22.0. IBM Corp., Armonk, NY, United States). Qualitative data were expressed in percentages (%) whereas quantitative variables were expressed in means and standard deviations. Linear regression analysis was used, and β coefficients were calculated to estimate the strength of relationship among daily sun exposure, daily Vit D intake, and serum 25(OH)D level. Logistic regression analysis was used to predict the odds for adequate serum 25(OH)D level (defined as ≥50 nmol/L in this study) in different sun exposure time and Vit D intake score groups. *p*-Values of less than 0.05 were regarded as statistically significant. Three expanded models are provided in each analysis to eliminate effects of nuisance variables: Model 1 = unadjusted; Model 2 = adjusted for age, gender, and race or ethnicity; Model 3 = Model 2 + adjusted for body mass index, serum glucose level, serum alanine aminotransferase level, serum creatinine level, and smoking history.

## Results

### Characteristics of Study Population

Demographic information of the participants is provided in Table 2. The average age was 49 ± 6 years. Average total serum 25(OH)D) level was 63 ± 26 nmol/L. Average daily sun exposure time and Vit D intake were 126 ± 115 min and 12 ± 21 mcg, respectively. Men compose 50.4% (*n* = 1,190) of the population, and non-Hispanic White accounts for the largest percentage of the population (*n* = 1,011, 43%).

**TABLE 2:** Characteristics of participants.

Characteristics	Total participants (*N* = 2,360)
**Continuous variables**	
Age(years)	49 ± 6
BMI(kg/m^2^)	30 ± 7
Serum glucose level (mg/dL)	103 ± 39
Serum alanine aminotransferase level (U/L)	27 ± 19
Serum creatinine level (mg/dL)	0.8 ± 0.4
Total serum 25-hydroxyvitamin D level (25(OH)D) (nmol/L)	63 ± 25
25-hydroxyvitamin D2 level (25(OH)D_2_) (nmol/L)	3 ± 10
25-hydroxyvitamin D3 level (25(OH)D_3_) (nmol/L)	60 ± 25
3-epi-25-Hydroxyvitamin D3 level (epi-25OHD_3_) (nmol/L)	4 ± 3
Daily sun exposure time(min)	126 ± 115
Daily vitamin D(D2 + D3) intake(mcg)	12 ± 21
**Categorical variables**	
Gender	
Male	1,190(50%)
Female	1,170(50%)
Race	
Mexican American	403(17%)
Other Hispanic	260(11%)
Non-Hispanic White	1,011(43%)
Non-Hispanic Black	449(19%)
Other Race – Including Multi-Racial	237(10%)
Smoking history	1,056(45%)

*BMI, body mass index. Values in the continuous variables were expressed as mean and standard deviation. Values in the categorical variables were expressed in number and percentage (%).*

### Associations Between Daily Sun Exposure Time, Daily Vitamin D Intake, and Serum 25(OH)D Level

Positive correlations were revealed between daily sun exposure time and serum 25(OH)D level (Table 3) either when calculated as continuous variable (β coefficient = 0.014, 95% CI = 0.005–0.024, *p*-value = 0.002) or score of 10 (β coefficient = 0.8, 95% CI = 0.4–1.2, *p*-value < 0.001). Similarly, positive associations were observed between daily Vit D intake and serum 25(OH)D level either when calculated as continuous variable (β coefficient = 0.4, 95% CI = 0.3–0.4, *p*-value < 0.001) or score of 10 (β coefficient = 3.0, 95% CI = 2.7–3.3, *p*-value < 0.001).

**TABLE 3:** Linear regression analyses for the associations between daily sun exposure time, daily vitamin D intake, and serum 25(OH)D level.

		Model 1	Model 2	Model 3
Daily sun exposure time(min)	β coefficient (95% CI)	0.012 (0.004,0.021)	0.018 (0.008, 0.027)	0.014 (0.005, 0.024)
	*P*-value	0.006	<0.001	0.002
Score of sun exposure time	β coefficient (95% CI)	0.7 (0.4, 1.1)	0.9 (0.6, 1.3)	0.8 (0.4, 1.2)
	*P*-value	<0.001	<0.001	<0.001
Daily vitamin intake(mcg)	β coefficient (95% CI)	0.4 (0.4, 0.5)	0.4 (0.4, 0.5)	0.4 (0.3, 0.4)
	*P*-value	<0.001	<0.001	<0.001
Score of vitamin D intake	β coefficient (95% CI)	3.0 (2.7, 3.4)	3.0 (2.7, 3.4)	3.0 (2.7, 3.3)
	*P*-value	<0.001	<0.001	<0.001

*Model 1 = unadjusted, Model 2 = adjusted for age, gender, and race/ethnicity, Model 3 = Model 2 + adjusted for body mass index, serum glucose level, serum alanine aminotransferase level, serum creatinine level, and smoking history.*

### Different Patterns of Sun Exposure and Vitamin D Intake Affecting Serum 25(OH)D Level

**FIGURE 1:**
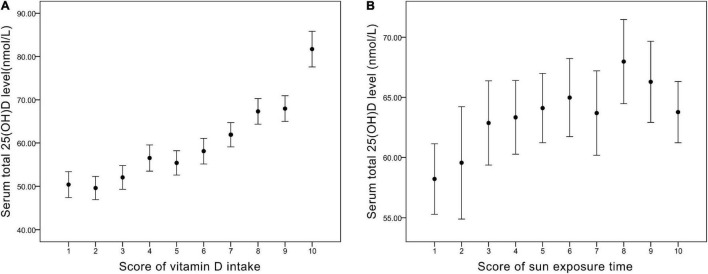
**(A)** The corresponding value of serum total 25(OH)D level (nmol/L) in each score of vitamin D intake. **(B)** The corresponding value of serum total 25(OH)D level (nmol/L) in each score of sun exposure time.

From Figure 1 and Table 4, we found that the variables were related in different manners. For Vit D intake, a steady rise in serum 25(OH)D level was observed (Figure 1), and odds ratio (OR) for adequate serum Vit D level increased along with the ascending scores (Table 4). On the other hand, a continued increase in serum 25(OH)D level was observed in sun exposure time score <5, whereas a plateau of serum 25(OH)D level around 65 nmol/L was reached in sun exposure time score >5 (Figure 1). OR for adequate serum Vit D level revealed similar pattern, with scores 1–5 increased from OR = 1.1 to 1.9, but scores 6–10 fluctuate between OR = 2.0 to 2.5. In brief, increase in Vit D intake brings about steady rise in serum 25(OH)D level, whereas increase of sun exposure time reaches a plateau of serum 25(OH)D level, echoing findings in the previous literature (36, 37).

**TABLE 4:** Logistic regression analyses for the associations between scores of sun exposure time, scores of vitamin D intake, and serum 25(OH)D level.

		Model 1	Model 2	Model 3
**Score of sun exposure time**				
1	OR (95% CI)	Ref.	Ref.	Ref.
	*P*-value	Ref.	Ref.	Ref.
2	OR (95% CI)	1.0 (0.69, 1.6)	1.2 (0.8, 1.8)	1.1 (0.7, 1.7)
	*P*-value	0.90	0.52	0.71
3	OR (95% CI)	1.5 (1.1, 2.1)	1.6 (1.2, 2.3)	1.6 (1.1, 2.2)
	*P*-value	0.023	0.006	0.012
4	OR (95% CI)	1.5 (1.1, 2.2)	1.6 (1.2, 2.3)	1.6 (1.1, 2.2)
	*P*-value	0.013	0.006	0.012
5	OR (95% CI)	1.9 (1.3, 2.6)	2.0 (1.4, 2.9)	1.9 (1.3, 2.7)
	*P*-value	<0.001	<0.001	<0.001
6	OR (95% CI)	1.8 (1.3, 2.6)	2.0 (1.4, 2.9)	2.0 (1.4, 2.9)
	*P*-value	0.001	<0.001	<0.001
7	OR (95% CI)	2.2 (1.4, 3.3)	2.3 (1.5, 3.6)	2.2 (1.4, 3.4)
	*P*-value	<0.001	<0.001	<0.001
8	OR (95% CI)	2.0 (1.4, 2.9)	2.2 (1.5, 3.1)	2.0 (1.4, 2.9)
	*P*-value	<0.001	<0.001	<0.001
9	OR (95% CI)	2.4 (1.6, 3.7)	2.6 (1.7, 3.9)	2.5 (1.6, 3.8)
	*P*-value	<0.001	<0.001	<0.001
10	OR (95% CI)	1.9 (1.4, 2.7)	2.0 (1.4, 2.8)	2.0 (1.3, 2.6)
	*P*-value	<0.001	<0.001	0.001
**Score of vitamin D intake**				
1	OR (95% CI)	Ref.	Ref.	Ref.
	*P*-value	Ref.	Ref.	Ref.
2	OR (95% CI)	1.1 (0.7, 1.5)	1.1 (0.7, 1.5)	1.1 (0.8, 1.7)
	*P*-value	0.77	0.74	0.50
3	OR (95% CI)	1.3 (0.9, 1.8)	1.2 (0.9, 1.8)	1.3 (0.9, 1.9)
	*P*-value	0.22	0.25	0.16
4	OR (95% CI)	1.8 (1.3, 2.6)	1.7 (1.2, 2.5)	1.8 (1.2, 2.6)
	*P*-value	0.001	0.004	0.002
5	OR (95% CI)	1.6 (1.1, 2.2)	1.5 (1.0, 2.2)	1.6 (1.1, 2.3)
	*P*-value	0.018	0.034	0.015
6	OR (95% CI)	1.8 (1.3, 2.6)	1.7 (1.2, 2.5)	1.8 (1.2, 2.6)
	*P*-value	0.001	0.004	0.002
7	OR (95% CI)	2.6 (1.8, 3.7)	2.5 (1.7, 3.6)	2.6 (1.7, 3.8)
	*P*-value	<0.001	<0.001	<0.001
8	OR (95% CI)	3.8 (2.6, 5.6)	3.8 (2.6, 5.7)	3.9 (2.6, 5.8)
	*P*-value	<0.001	<0.001	<0.001
9	OR (95% CI)	4.2 (2.4, 6.3)	4.5 (3.0, 6.7)	4.8 (3.2, 7.3)
	*P*-value	<0.001	<0.001	<0.001
10	OR (95% CI)	7.6 (4.8, 11.9)	8.5 (5.4, 13.4)	9.3 (5.8, 14.7)
	*P*-value	<0.001	<0.001	<0.001

*Model 1 = unadjusted, Model 2 = adjusted for age, gender, and race/ethnicity, and Model 3 = Model 2 + adjusted for body mass index, serum glucose level, serum alanine aminotransferase level, serum creatinine level, and smoking history.*

### The Impact of Sun Exposure Time for Low-Score Groups of Vitamin D Intake

**FIGURE 2:**
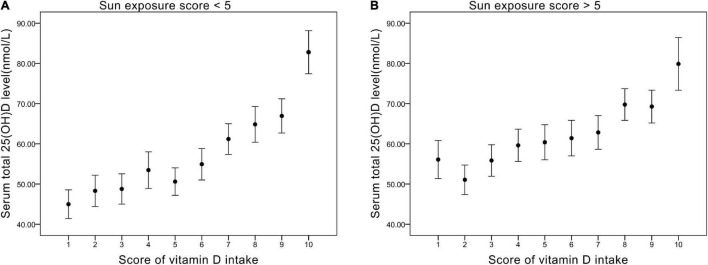
**(A)** The relationship between vitamin D intake score and serum total 25(OH)D level (nmol/L) in groups of sun exposure score <5. **(B)** The relationship between vitamin D intake score and serum total 25(OH)D level (nmol/L) in groups of sun exposure score >5.

We divided sun exposure time into two parts — sun exposure time score <5 (relatively low exposure group) and sun exposure time score >5 (relatively high exposure groups) — and plotted their relationship with Vit D intake score, respectively (Figure 2). For scores 1–5 of Vit D intake, relatively high sun exposure (Figure 2B) gave rise to higher serum 25(OH)D levels (average 57 nmol/L) compared with relatively low sun exposure (Figure 2A) (average 50 nmol/L). On the contrary, as Vit intake increased (score 6–10), the difference in serum 25(OH)D level between sun exposure score >5 and <5 became negligible (both ranged from 60 to 80 nmol/L). Collectively, the impact of sun exposure time on serum 25(OH)D level is more noticeable in the relatively low Vit D intake groups.

### Collaborative and Individual Contribution of Vitamin D Intake and Sun Exposure Time to Serum 25(OH)D Level

**FIGURE 3:**
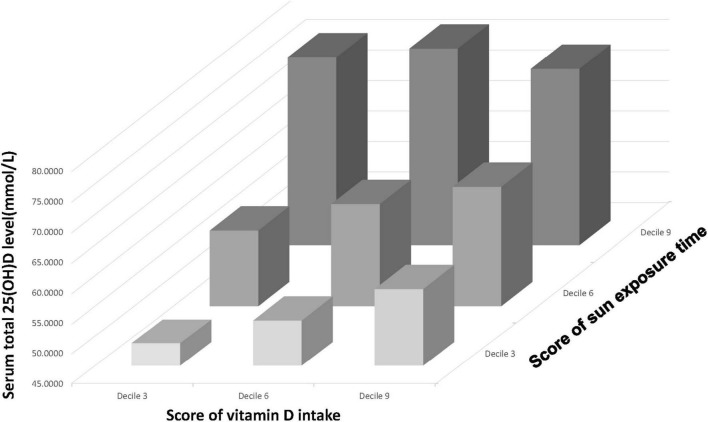
A three-dimensional histogram that plots the relationship between Vit D intake (presented on the *x*-axis), sun exposure time (presented on the *y*-axis) and serum 25(OH)D level (presented on the *z*-axis).

A three-dimensional histogram was plotted to illustrate the collaborative and individual contributions of Vit D intake and sun exposure time to serum 25(OH)D level (Figure 3). Generally, the increase in Vit D intake and sun exposure time both led to an increase in serum 25(OH)D level. However, a deep investigation would unveil the discrepancies between the two. The increase in sun exposure yielded a steady increase in serum 25(OH)D level in deciles 3,6, and 9 of Vit D score. In contrast, the increase in Vit D intake brought the most significant rise to serum 25(OH)D level in decile 3 of sun exposure time, and less degree of rise in decile 6, whereas the least degree of change in decile 9. Figure 3 represented the unadjusted population, and the adjusted populations (data not shown) followed similar trends. Taken together, when receiving relatively high sun exposure, the impact of Vit D intake on serum 25(OH)D level becomes trivial.

### Interactions Between Vitamin D Intake and Sun Exposure Time

**FIGURE 4:**
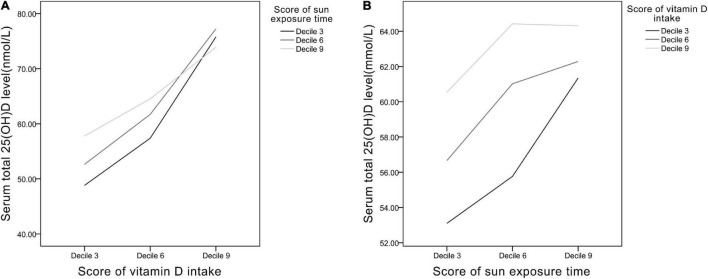
The relationships between **(A)** Vit D intake and **(B)** sun exposure time presented as scores of deciles 3, 6, and 9.

Line graphs were plotted to evaluate the interactions between Vit D intake and sun exposure time (Figure 4). In Figure 4A, in the low Vit D intake group (decile 3 of Vit D intake score), the increase of sun exposure can significantly induced rise in serum 25(OH)D level, whereas in the high Vit D intake group (decile 9 of Vit D intake score), the increase of sun exposure brought negligible changes in serum 25(OH)D. In Figure 4B, the greatest slope occurred in the low Vit D intake curve (decile 3 of Vit D intake), which shows increased sun exposure time causing the greatest degree of change in serum 25(OH)D level in this group. Collectively, in the relatively low Vit D intake group, increase of sun exposure can elevate serum 25(OH)D level noticeably, whereas in the relatively high Vit D intake group, moderate sun exposure time results in adequate serum 25(OH)D level.

### Serum 25(OH)D Level in Specific Populations

**FIGURE 5:**
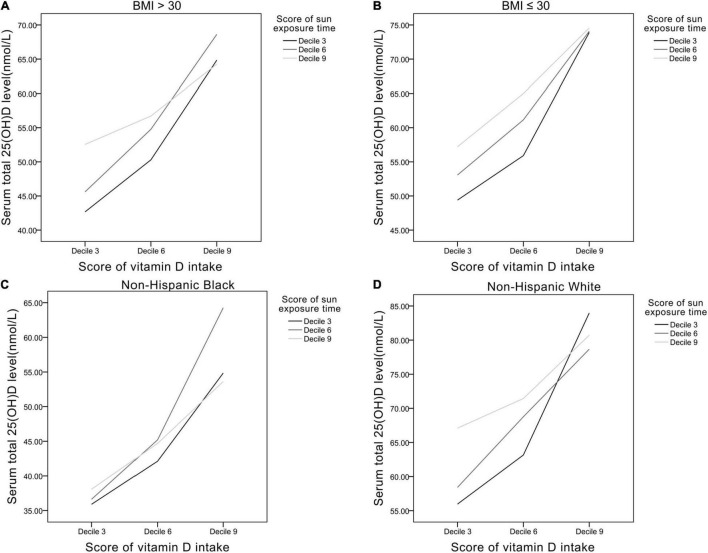
The relationships between Vit D intake and sun exposure time in panel **(A)** BMI > 30 group, **(B)** BMI ≤ 30 group, **(C)** non-Hispanic black, and **(D)** non-Hispanic white.

Analyses in specific populations were performed (Figure 5). In obese participants (BMI > 30), average serum 25(OH)D level was lower (43–68 nmol/L) than the non-obese participants (BMI ≤ 30) (50–74 nmol/L). In non-Hispanic Black, average serum 25(OH)D level was also lower (36–64 nmol/L) compared with non-Hispanic White (55–84 nmol/L).

## Discussion

This study highlights the essential role of sun exposure in Vit D production. Moderate sun exposure can significantly make up for deficiencies in subjects who consume relatively low dietary Vit D. From another perspective, dietary Vit D supplement in subjects receiving relatively high sun exposure is not a demanding need.

In our study, the average serum 25(OH)D) level was 63 ± 26 nmol/L, but the average daily Vit D intake was 12 ± 21 mcg, which implies that 50% of healthy individuals reached sufficient serum 25(OH)D) level (>50 nmol/L) but did not satisfy the RDA of Vit D (>15 mcg/day in ages 1–70 years) provided in the Dietary Reference Intakes (DRIs) developed by expert committees of NASEM (38) or the dose for adults (>600 IU/day, which equals 15 mcg/day) suggested in clinical practice guideline of The Endocrine Society (39). This is consistent with an analysis of What We Eat in America, NHANES 2015-2016 (40). They reported that most population (82%) who ingested vitamin D only from food and beverages achieved an average of 4.8 mcg/day of dietary Vit D intake, whereas minor proportion (28%) who took dietary Vit D supplement could achieve an average of 19.9 mcg/day of dietary Vit D intake. The proportion of supplement users is relatively low, which implies that public awareness is lacking and affordability in specific groups is worrying, and echoes previous findings in US civilians (41).

On the other hand, over 50% healthy individuals were considered Vit D sufficient while not meeting the recommended dietary goal, then who plays the key role within? Findings in our study illustrate that sun exposure can significantly improve serum 25(OH)D levels in individuals who ingested relatively low Vit D. In other words, for those who are unable to obtain sufficient dietary Vit D (may be due to inaccessibility, financial constraints, or dietary habits), basking in the sun in moderation appears to be a good alternative. We further estimated the cutoff point of sun exposure time to achieve sufficient serum 25(OH)D level in our participant with receiver operating characteristic analysis, and the result revealed 35 min/day (*p* < 0.001) was the optimal timepoint (sensitivity: 78.5%, specificity: 32.9%), which is also in line with the agreement by most experts (18–20, 42, 43). In fact, experts have advocated that the aforementioned amount of sun exposure is enough to make 1,000 IU Vit D/day, which is equivalent to 25 mcg/day of Vit D intake (44). The easily achieved length of time without costing a penny may encourage more individuals to put this into daily practice.

The efficacy of improving serum 25(OH)D by UVB exposure has been demonstrated in numerous studies. A study of elderly female patients in nursing home in the Netherlands showed a significant increase in 25(OH)D, from a median of 18 to 60 nmol/L, in the experimental group that received UVB radiation (0.5 minimal erythemal dose) to the lower back three times per week during 12 weeks (45). Another study recruiting subjects with skin disease from Scotland measured an increase in serum 25(OH)D from 34 to 78 nmol/L after 4 weeks of narrow band UVB treatment (46). A recent study provided a practical sun exposure regime for white Caucasians at UK latitudes and advocated that short (9 min a day), specific timing (sunlight at lunchtime from March to September) exposure is already enough to remain adequate serum Vit D level throughout the winter (47). Collectively, low-dose, repeated exposure at correct timing permits Vit D adequacy in white-skinned individuals (37). Regimes may differ in other races, but comparable dose–response characteristics of Vit D synthesis in skin can be anticipated.

Health disparity of Vit D inadequacy is possibly contributed to socioeconomic differences. Studies have shown several characteristics in low socioeconomic status related to higher risk of Vit D deficiency. Education level may reflect the awareness for health behaviors such as alcohol abstinence, smoking cessation, and outdoor activity levels which influence the production of Vit D (48, 49). Furthermore, less prudent dietary habits and the unaffordability of additional Vit D supplementation are also the unfavorable factors for Vit D level (50). In view of this, the encouragement of increasing sun exposure time may be a relatively simple and practical means for vulnerable populations.

Putting forward this argument brings along several noteworthy issues. First, risk-benefit analysis of sun exposure to strike a balance between optimal cutaneous Vit D production and skin diseases has long been a debate. However, as maximum cutaneous Vit D production occurs rapidly within a brief sun exposure time (42), the risk for photodamage is minimized (51, 52). Besides, several recent studies rebut the relationship between sun exposure and skin cancer, which suggest that the issue requires more evidence to establish the causality (53, 54). Second, aside from Vit D synthesis, solar exposure brings plenty of health benefits. UV radiation modulates nitric oxide bioactivity in circulation and thus bring beneficial cardiovascular effects (11, 55). Metabolic dysfunction such as overweight, high low-density lipoprotein cholesterol level, and abnormal insulin levels is reduced in mice irradiated with UV light (56). Inverse relationship is observed between sun exposure and all-cause mortality (9, 57). Circadian clocks regulating behavior, physiology, and metabolism can be disrupted by insufficient sun exposure (58). Third, in groups with inferior abilities of cutaneous Vit D synthesis, such as the dark-skinned (59), the obese (60), and the older adults (61), or people living at high latitudes who are unable to obtain sufficient sun exposure (62), the importance of dietary supplements or fortified foods containing Vit D should still be emphasized (14).

There may be some limitations in this study. First, there is still no global consensus on definition of Vit D inadequacy. However, the trend of insufficient Vit D intake is common worldwide (63, 64), and thus, the practice of moderate sun exposure may be a solution to the problem. Second, the same sun exposure time in different geographical locations may result in widely differing doses of UVB. Since sun exposure time was the only available data we could approach in NHANES, future studies adopting accurate UVB doses are needed to preform precise analyses. Third, NHANES provides representative data of healthy US civilians, and hence, the generalizability to different races, regions, and individuals with specific diseases is limited. The majority of subjects were white-skin ethnicity, which may underestimate the challenges for dark-skin individuals to achieve the same adequate Vit D level. Studies have demonstrated that melanin in skin blocks UV radiation that is necessary for Vit D synthesis (59) and called for the higher need of diet fortification or supplementation in dark-skin individuals (65, 66).

## Conclusion

Vitamin D inadequacy is associated with multiple adverse health outcomes and therefore deserves public awareness. In view of the common insufficiency of dietary Vit D, we demonstrated that increasing sun exposure can significantly make up for the deficiencies. Compared with the extra cost and time for building habits of Vit D supplementation, sun exposure for a short 30 min every day seems to be a simpler practice to start with. Implementation of sun exposure also reduces the demand for dietary supplements, which eases the situation in another way. Promotion of moderate sun exposure may turn out to be an effective means of mitigating prevalence of Vit D inadequacy.

## Data Availability Statement

The datasets presented in this study can be found in online repositories. The names of the repository/repositories and accession number(s) can be found below: https://www.cdc.gov/nchs/nhanes/index.htm.

## Ethics Statement

The studies involving human participants were reviewed and approved by the National Health and Nutrition Examination Survey. The patients/participants provided their written informed consent to participate in this study.

## Author Contributions

S-EW drafted the manuscript. W-LC conceptualized and designed the study, supervised all aspects of the study, critically reviewed and revised the manuscript, and approved the final manuscript as submitted. Both authors contributed equally to the literature search, study design, data collection and data interpretation, and meet the ICMJE criteria for authorship.

## Conflict of Interest

The authors declare that the research was conducted in the absence of any commercial or financial relationships that could be construed as a potential conflict of interest.

## Publisher’s Note

All claims expressed in this article are solely those of the authors and do not necessarily represent those of their affiliated organizations, or those of the publisher, the editors and the reviewers. Any product that may be evaluated in this article, or claim that may be made by its manufacturer, is not guaranteed or endorsed by the publisher.

## Footnotes

1
https://www.cdc.gov/nchs/nhanes/index.htm

2
http://www.ars.usda.gov/ba/bhnrc/fsrg
